# Relative Contribution of Proprioceptive and Vestibular Sensory Systems to Locomotion: Opportunities for Discovery in the Age of Molecular Science

**DOI:** 10.3390/ijms22031467

**Published:** 2021-02-02

**Authors:** Turgay Akay, Andrew J. Murray

**Affiliations:** 1 Atlantic Mobility Action Project, Brain Repair Centre, Department of Medical Neuroscience, Life Science Research Institute, Dalhousie University, Halifax, NS B3H 4R2, Canada; 2 Sainsbury Wellcome Centre for Neural Circuits and Behaviour, University College London, London W1T 4JG, UK

**Keywords:** somatosensory feedback, vestibular feedback, locomotion, speed, molecular sciences

## Abstract

Locomotion is a fundamental animal behavior required for survival and has been the subject of neuroscience research for centuries. In terrestrial mammals, the rhythmic and coordinated leg movements during locomotion are controlled by a combination of interconnected neurons in the spinal cord, referred as to the central pattern generator, and sensory feedback from the segmental somatosensory system and supraspinal centers such as the vestibular system. How segmental somatosensory and the vestibular systems work in parallel to enable terrestrial mammals to locomote in a natural environment is still relatively obscure. In this review, we first briefly describe what is known about how the two sensory systems control locomotion and use this information to formulate a hypothesis that the weight of the role of segmental feedback is less important at slower speeds but increases at higher speeds, whereas the weight of the role of vestibular system has the opposite relation. The new avenues presented by the latest developments in molecular sciences using the mouse as the model system allow the direct testing of the hypothesis.

## 1. Introduction

Locomotion is a fundamental animal behavior that is necessary for survival and, consequently, has been a strong focus for research in life sciences. In most terrestrial animals, from insects to mammals, locomotion is achieved by moving multiple multi-segmented appendages, the legs, in a rhythmic and coordinated fashion [1,2]. Current advances in molecular sciences have presented unprecedented opportunities to investigate the neuronal mechanisms underlying this intra-leg and the inter-leg coordination. In particular the mouse model, with its combination of relatively complex behavior and neural circuit access via molecular–genetic methods, has emerged as a key tool in our quest to understand terrestrial locomotion [3,4,5,6].

Though central neuronal circuits, and even the isolated spinal cord alone, can generate the basic locomotor rhythm, sensory feedback plays a crucial role in the production of coordinated and goal-directed locomotion. In this review, we will briefly outline our current understanding on the neuronal control of locomotion with a specific focus on the role of sensory feedback from two sources. First, that from the legs (segmental somatosensory feedback) signaling touch, movement or position of the leg or the force. Second, sensory feedback from the inner ear (vestibular feedback) that signals the rotation and acceleration of the head. We will outline why we believe the respective roles of proprioceptive and vestibular feedback are dependent on locomotor speed, vestibular feedback being critical at lower speeds and somatosensory feedback is necessary at higher velocities. Finally, we will discuss how modern molecular–genetic techniques provide exceptional possibilities to further understand the locomotor circuitry.

### 1.1. Intra-Leg Coordination during Locomotion

Intra-leg coordination, i.e., coordination of the movement of a single limb, involves movement in multiple joints. In mammals, the coordinated movement of the three main joints (hip, knee and ankle) of a leg is achieved by the temporally constrained contractions of several dozen muscles [1,4]. These coordinated muscle contractions are controlled by pools of individual muscle-dedicated motor neurons located in the cervical or lumbar enlargements of the spinal cord for the fore or the hind limbs, respectively [7]. The activity of these motor neuron pools is driven by a complex network of premotor interneurons that make up the central pattern generator (CPG) [4,7]. The CPG works in collaboration with sensory feedback from the leg (segmental somatosensory) [2,8] or the supraspinal centers (e.g., visual or the vestibular) [9,10] to generate a locomotor pattern that is flexible enough to deal with obstacles or unpredictable changes in the terrain.

When we consider the rhythmic movement of an individual leg, we recognize two stages that make up one step: the stance and the swing phase. The foot is on the ground during the stance phase and moves in the opposite direction of locomotion with respect to the body and provides body support and propulsion. When the leg is extended to a certain degree, the foot lifts off the terrain and moves in the direction of locomotion to be placed back on the ground and start the next stance phase [1,2]. This overall structure of the step is similar at all speeds of walking, but the relative duration of the stance phase as a portion of a step cycle (the duty cycle) is modulated as the speed changes during locomotion [1,11].

### 1.2. Inter-Leg Coordination during Locomotion

Inter-leg coordination involves organization of the movement of multiple legs. Spinal commissural pathways are in place to maintain the coordination of legs on the left and right sides, and propriospinal pathways coordinate legs of different segments [12,13,14]. The main goal here is for animals with multiple legs to maintain an area of body support that surrounds the extrapolated center of the body mass or places the feet in front of the extrapolated center of mass during running gaits to avoid destabilization and falls [15,16]. This is achieved by diverse interlimb coordination patterns such as walking–trotting–galloping–bounding in quadrupeds [17], walking–running in bipedal humans [18] or tripod and tetrapod pattern in insects [19,20].

In quadrupedal mammals, when locomoting at slow speeds, walking is the preferred gait, where the swing phases of the left and right legs alternate with each other (they move in antiphase). Moreover, during walking, the hind and front legs of the same side swing temporally closer to each other but do not overlap. The overlap of the swing movements (in phase) of homolateral legs occurs in a relatively uncommon gait called pacing [21], which will not be discussed here. During trotting, which occurs at slightly faster locomotion speeds, not only are the left and right legs in antiphase, but the hind and front legs also swing in antiphase, causing the diagonal legs to swing in synchrony. At higher speeds, the left and right leg swing movements start to overlap (in phase), leading to the galloping gait. Finally, at the fastest locomotion speed, the swing movements of the left and hind legs are synchronous, a gait that is called bounding [17]. In bipedal humans, the gait for the slowest locomotor speed is walking, when at least one foot is on the ground at all times. At faster speeds, the locomotor gait changes to running, where there are periods with both feet in the air. Faster running may also be classified as sprinting, though it is unclear whether this is a distinct gait. Mechanically, the main difference between walking and running is the way animals use their kinetic and potential energy in the most efficient way to reduce the work needed to accelerate and maintain the desired speed [22,23,24]. In hexapod locomotion in insects, the animals have a quadrupedal coordination pattern at their slowest speed, which transitions gradually into a tripodal coordination pattern as their speed increases [19,20]. Research in animal models indicate that segmental somatosensory feedback [25,26] and feedback from the vestibular system [27] might underlie the correct coordination pattern during locomotion, but the details of how these two feedback patterns play a role at different speeds is not understood.

In this review, we sought to summarize the research investigating the role of segmental somatosensory feedback and the feedback from the vestibular system during locomotion at different speeds in mammals. This review will lead to the hypothesis that the weight of the role of segmental feedback is less important at slower speeds but increases at higher speeds, whereas the weight of the role of the vestibular system has the opposite relation.

## 2. Role of Segmental Somatosensory Sensory Feedback in Locomotion

### 2.1. Overview on Segmental Sensory Feedback on Locomotion

A major source of the sensory feedback during locomotion comes from the segmental afferents that signal the current position, movement and force of the body, collectively called proprioception and signals originating from the external world, referred to as exteroception [28]. Proprioceptive information is mainly transmitted by myelinated Group Ia and Group II afferents from the muscle spindles and the Group Ib afferents from the Golgi tendon organs, respectively [29,30]. The signals provided by the Group Ia/II afferents from the muscle spindles are related to muscle stretch and are therefore an indirect measurement of the angular displacement of individual joints. On the other hand, the signals conveyed by the Group Ib afferents relate to tension in the tendons and therefore measure the force or load. Experiments on human subjects suggest that touch and stretch-sensitive cutaneous afferent feedback also contributes to the sensation of joint movements [31,32,33,34]. The stimuli that are related to proprioception therefore originate from one’s own body posture or movement. Exteroceptive information is conveyed by a large array of cutaneous afferents coming from the skin cutaneous receptors that signal skin deformation due to touch, stretch, vibration, pressure (mechanoreception), temperature (thermoception) or stimuli perceived as painful (nociception) [35,36]. The common aspect of these exteroceptors is that the stimuli originate from outside of the body. Different aspects of locomotor movements are influenced by either of these feedback modalities; we outline how below.

**Figure 1:**
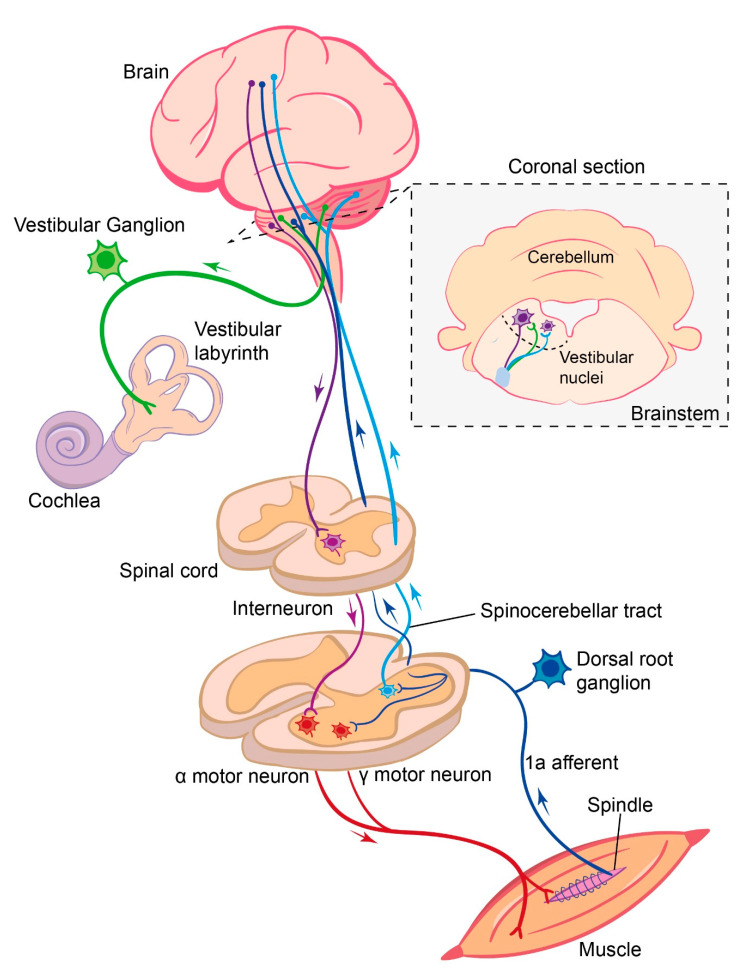
Summary of the somatosensory and vestibular sensory pathways and their integration into the brain and spinal cord.

The cell bodies of segmental sensory afferent neurons are located in the dorsal root ganglia adjacent to the spinal cord. From this cell body, a single neuronal process emerges that further diverges into a peripheral and a central process (giving these sensory neurons a monopolar neuron structure) (Figure 1). The peripheral branch of the proprioceptive afferent neurons project out to the muscles to innervate either the muscle spindles, as for the Group Ia and II afferents, or the Golgi tendon organs, as for the Group Ib afferents, whereas the peripheral processes of the cutaneous afferents innervate the skin [37,38,39]. The central branch enters the spinal cord through the dorsal root entry zone and forms synapses with diverse inter- or motor neurons located in the grey matter of the spinal cord [40,41,42]. While some of these interneurons are involved in information processing within the spinal cord, others carry the sensory information further to the brain through specific pathways, such as the dorsal and ventral spinocerebellar pathways [43,44] and the spinothalamic tracts [39,45]. In addition to these second-order afferents, a branch of the primary afferents, conveying proprioceptive information, projects up to the supraspinal centers through the dorsal column of the white mater [39]. Of all the afferent fibers entering the spinal cord, only the Group Ia afferents’ central projections makes direct synaptic contact with the motor neurons [46,47]. A number of these pathways are summarized in Figure 1.

### 2.2. Exteroceptive Sensory Feedback and Locomotion

Research conducted in early 20th century demonstrated that the removal of cutaneous feedback in an otherwise intact cat does not cause significant changes in its walking behavior, suggesting that exteroceptive sensory feedback is not necessary for locomotion [48]. These observations were confirmed in later studies showing that the removal of cutaneous feedback caused only minor, transient changes in locomotion on a flat terrain, and these effects diminished in a matter of weeks [49]. However, significant changes were observed when descending information from the brain was also missing in addition to the absence of the cutaneous feedback [50]. In more recent studies, it has been shown that cutaneous feedback is important in maintaining lateral stability during walking on a split-belt treadmill [51]. The role of cutaneous feedback becomes more apparent when cutaneous afferent fibers are experimentally stimulated during ongoing walking. For example, the stimulation of cutaneous afferents from the plantar surface of the foot during walking causes either an increase or initiation of the extensor muscle activity, depending on the phase of the step cycle during stimulation [52]. Interestingly, a stimulation of cutaneous afferents from the dorsal surface of the foot either augments the extensor activity if the stimulation occurs during a stance or causes flexor muscle activity if the stimulation occurs during a swing phase [53,54]. The latter flexor response to dorsal foot stimulation is called the “stumbling corrective reaction”, as it is a reflex response that initiates a higher swing movement to clear obstacles hitting the dorsal side of the food while walking [55]. Overall, it appears that cutaneous feedback is essential for the fine control of locomotion, though its loss can be at least partially compensated for by the supraspinal centers.

### 2.3. Segmental Sensory Feedback and Posture

The general physical rules dictate that, for a stable posture in terrestrial animals, the center of mass must be kept within the base of support during standing [15]. Due to the dynamic conditions of locomotion—that is, the forward and lateral movements of the body—a modification has been proposed that incorporates the velocity of locomotion and length of the limb into the center of mass position [16]. However, does segmental somatosensory feedback play a role in maintaining this stable posture during movement? Past research in cats suggests that cutaneous feedback is important to maintaining stability during locomotion, especially in the presence of external perturbations [26,51,56,57,58]. A critical role of cutaneous feedback in the regulation of the center of mass has also been suggested in human experiments [59]. Furthermore, muscle spindles have been shown to provide information regarding the direction and velocity of perturbations, which is critical for maintaining stability in dynamic environments [60]. In accordance with this, proprioceptive feedback has been shown to be important to maintaining stability in humans [61,62,63]. These results suggest that segmental sensory feedback is necessary for a stable posture during standing and walking.

Ensuring proper posture, with the center of mass maintained within the base of support, in an irregular environment requires sensory feedback to coordinate the position and movement of multiple legs. A very successful historical overview of these reflexes was presented by [64]. The most intensively investigated interlimb reflex are the crossed reflexes, where the stimulation of somatosensory afferents of one leg causes a motor response in the contralateral legs. This reflex has been described in cats [48,65,66,67], rodents [68,69,70] and humans [71,72]. Moreover, using animal models, commissural interneurons have been identified that are involved in transmitting somatosensory information to the contralateral side of the spinal cord [40,73]. Besides the crossed reflexes, sensory influences that coordinate the activity of the hind and fore legs have also been demonstrated in cats [26] and rats [12]. Significant progress is underway in order to understand the neuronal circuitry that coordinates the activity between the legs and, therefore, maintains a stable posture during standing and walking, despite perturbations.

### 2.4. Proprioceptive Sensory Feedback and Locomotion

In contrast to exteroceptive feedback, proprioceptive feedback is required for normal stepping behavior with a loss of proprioception due to diseases in humans or experimental animals having a detrimental effect on locomotion [74,75,76]. The loss of proprioceptive feedback in humans with a rare form of large fiber neuropathy causes severe deficits in movement and locomotion, unless these patients learn to compensate for the loss of proprioceptive feedback with vision [76]. It has also been shown using animal models that the removal of proprioceptive feedback, either along with all other types of afferent feedback using surgical methods [77,78,79] or selectively using genetics [74,75] or chemical ablation [80], has a detrimental effect on the generation of the locomotor pattern. However, the effect on quadrupedal animals seems to be less severe than in bipeds. The reason for the milder effect in animals such as mice is presumably due to the more stable postures of quadrupeds vs. bipeds due to the lower center of mass and increased base of support [81]. Moreover, if proprioceptive feedback from the muscle spindles is missing but the feedback from the Golgi tendon organs remains intact, the effect is less severe and more prominent in the swing phase of the step cycle [74]. These observations suggest that normal locomotion in a natural environment requires proprioceptive feedback.

Is the proprioceptive influence on the locomotor pattern similar at all speeds, or is this type of feedback more predominant during certain velocities? The H-, or Hoffmann, reflex provides a potential means of examining the strength of proprioceptive feedback during behaviors [82,83]. Here, a stimulation of the peripheral nerve is propagated to the spinal cord, where the synaptic actions on motor neurons can be read out as EMG recordings from the muscles. The strength of this reflex can be modified by changes in the central circuitry, such as alpha-motor neuron excitability or the presynaptic inhibition of proprioceptive sensory terminals. The H-reflex decreases in gain during running compared to walking in humans [84], potentially suggesting a reduced proprioceptive feedback at faster speeds. However, animal investigations have shown that, in the absence of proprioceptive sensory feedback from the muscle spindles, mice do not locomote at faster speeds, suggesting proprioceptive feedback is required at higher velocities [11]. We see two possibilities that could reconcile these apparently contradictory results. First, the human studies compared H-reflex across two distinct gaits, running and walking, whereas the mice maintained a trotting gait across a variety of speeds [11,84]. The second explanation concerns the route of proprioceptive feedback through the nervous system. The H-reflex measures excitability mainly at the proprioceptive-motor neuron synapse. The presynaptic inhibition of sensory afferents permits the nervous system to reduce an activity at a specific branch, while not affecting the other outputs of the same neuron [85]. Indeed, the H-reflex gain is reduced during behaviors where proprioceptive feedback should be critically important, such as in the absence of vision or when standing on an unstable surface [86]. It has been suggested that this downregulation of H-reflex gain occurs via presynaptic inhibition of the sensory neuron to motor neuron synapse and serves to attenuate the spinal stretch reflexes that could hinder balance [87]. The observation that presynaptic inhibition can attenuate local reflex responses and ascending information flow independently [85] suggests that individuals are protected against the loss of balance while preserving the awareness of limb positions. A similar mechanism could be at play during locomotion at different speeds.

Nevertheless, these observations do not mean that proprioceptive feedback is not required for slow locomotion, as many studies have demonstrated that the normal locomotor pattern is eroded in the absence of proprioceptive feedback [74,75]. A possible explanation is that an alternative mechanism might be able to compensate for the loss of proprioceptive feedback at slower speeds but not at faster speeds. We posit that this alternative mechanism is vestibular feedback, as it has been demonstrated that animals, including humans, with vestibulopathy avoid walking slower speeds [88,89], suggesting that vestibular feedback has a more significant role during slow walking than faster locomotor speeds.

## 3. Role of Vestibular Sensory Feedback in Locomotion

### 3.1. Vestibular Sensory Feedback

As the head moves through space, both rotation and linear acceleration are detected by organs in the vestibular labyrinth. Rotation is perceived by three bilateral, orthogonal semi-circular canals. These canals contain a viscous fluid, the endolymph, whose movement deflects hair cells, altering their activity [90]. Linear acceleration is detected in two planes (horizontal and vertical) by the otolith organs, where small grains known as otoconia move in response to acceleration and again deflect hair cells. In turn, sensory signals are transferred to the brain via vestibular sensory neurons that project to the brainstem and cerebellum.

How the central nervous system uses vestibular sensory information largely depends on two factors. (i) The type of sensory afferent that conveys vestibular signals and (ii) where in the nervous system those sensory afferents project. There are two types of vestibular sensory afferents, classified according to their discharge patterns in the absence of stimulation. These are regular and irregular afferents, with the two types also having differences in their anatomical, as well as physiological, properties [91]. Both types of afferent have a resting discharge that permits a bidirectional response to stimulations, i.e., a decrease in firing with hair cell deflection in one direction and an increase when deflected in the other. Regular, or tonic firing, afferents encode the angular head velocity (from the canals) and linear acceleration with respect to gravity (from the otoliths). Irregular, or phasic, afferents encode both the changes in head velocity and acceleration [92,93]. These sensory neurons are bimodal, with their cell bodies in the vestibular, or Scarpa’s, ganglion. The central branch of the vestibular nerve mostly terminates with the various nuclei of the vestibular nuclear complex. However, primary afferents also innervate the floccular–nodular lobe of the cerebellum, and there are reports that some fibers innervate non-vestibular nuclei of the brainstem, such as the cuneate and lateral reticular nucleus (Figure 1) [94].

In considering the vestibular contributions to locomotion, it is important to note that there are rarely “pure” vestibular signals found in the brain. As the vestibular end organs are located in the head and the head can be positioned on multiple planes on the body, the correct interpretation of vestibular signals also requires the immediate integration of proprioceptive information. Proprioception from the neck allows the nervous system to infer the position of the head on the body and then the direction of rotation or acceleration detected by the vestibular organs. Indeed, many second-order vestibular neurons (i.e., those that receive input from primary vestibular afferents) are concurrently innervated by proprioceptive afferents [95]. In turn, this combined sensory signal can influence multiple descending pathways that have access to spinal motor circuits. For example, multiple reticulospinal populations receive second-order vestibular sensory information [96]. Vestibulospinal populations also innervate the cervical and lumbar cord and may themselves play an important contribution in locomotion, though their close connection with the cerebellum means that it is challenging to infer how much of their output is mediated by vestibular afferents vs. higher order pathways [10,97].

The initial neural circuitry of the vestibular system is complex, with different afferent types encoding different angles and velocities of the head movement, which, in turn, is relayed to multiple regions in the brainstem and cerebellum. There are therefore multiple potential ways in which vestibular signaling could influence locomotion. Below, we outline some of the potential roles.

### 3.2. Maintaining Vision during Locomotion

A key behavioral process that requires the vestibular system is not directly related to the locomotor pattern itself but does facilitate the behavior. During locomotion, both the head and body are deflected in the vertical plain. The head can be stabilized on the body via the actions of the vestibulocollic reflex (VCR), generated by vestibular feedback. This reflex stabilizes the head on the body in response to locomotion and other movements [98]. Stabilization of the gaze itself is achieved by a complimentary reflex, the vestibular ocular reflex (VOR). The VOR uses vestibular afferent information, routed through the vestibular nucleus and floculus of the cerebellum, to directly control ocular motor neurons [99]. During locomotion, the actions of the VOR mean that visual acuity can be maintained to similar levels during walking and running as observed by standing in place [100]. Though most studies point to the role of the vestibular organs in the VOR, this reflex may be influenced by ascending circuits associated with the spinal central pattern generator [101]. Interestingly, the VOR may be the most effective at slow locomotor speeds, with a feedforward approach preferred at higher velocities [102].

### 3.3. Maintaining Stability and Balance

The most well-known function of the vestibular system is the maintenance of balance and stable posture. The vestibular system is required for maintaining balance during standing, particularly in humans and other bipeds [103]. Further, postural reflexes that respond to unexpected perturbations require a functioning vestibular system [104]. The vestibular system can therefore be considered as “stabilizing”, acting to counteract the effects of body movement, gravity and other external forces. This may seem counterintuitive to a role in locomotion; if the vestibular system wants to keep us in place, why would it be required for locomotion, which is inherently unstable? This problem was initially postulated by von Holst and Mittelstaedt [105]. Interestingly, vestibular reflexes have been shown to be downregulated when humans transition from standing to walking [106,107]—that is, its use is state-dependent. Initially, vestibular pathways are downregulated to allow gait initiation but are then utilized again during the double-support phase of walking [107]. This study points to a phase-specific role for vestibular pathways during locomotor behaviors, with the vestibular sensory information most predominant during the double-support phase of bipedal stepping. Whether similar mechanisms are found in more stable bipeds is not clear.

### 3.4. Vestibular Damage and Gait

Patients with damage to the vestibular system suffer from postural instability and an inability to appropriately respond to unexpected perturbations [108]. A central problem in ascribing a functional role to the vestibular system in locomotion is an inability to disambiguate the vestibular system’s role in maintaining balance and upright posture and the potential role in the generation of the locomotor pattern. That is, as we locomote, we must maintain our balance. So, is the vestibular system simply coordinating with other motor pathways to ensure that we maintain an upright posture during locomotion or does it have a fundamental role in generating the locomotor pattern itself? Though this is an interesting question from a circuit perspective, from the point of view of animal behavior, it may be a moot point. Given that it is impossible to generate natural locomotion in the absence of an upright posture, does the nervous system even consider these as two separate control problems?

Nevertheless, we can infer some functions of the vestibular system during locomotion by examining people and animals with either damage to the vestibular organs or by electrical stimulation of these organs. Damage or disruption to the vestibular system in humans can result from disorders such as vestibular neuritis or Meniere’s disease. The locomotor pattern in patents with peripheral vestibular damage is severely altered. Patients show an increased trunk sway, reduced step length, increased base of support, prolonged double-stance phase and increased variability [109]. At first glance, this phenotype would seem to indicate that the vestibular system plays multiple roles in the generation of the normal locomotor pattern. However, many of these can be considered as a secondary consequence of a loss of balance. Similarly, in quadrupedal animals with vestibular lesions, the main phenotypes are also associated with disruptions to the balance system. Animals generally maintain a lower center of mass, reduced cadence, shorter swing and variability in foot placement [110,111].

In humans, the primary vestibular afferents can be stimulated by galvanic vestibular stimulation, the application of an electrical current through the mastoid process, resulting in an increase in vestibular afferent activity on the side of the cathode, and a decrease on the side on the anode. During walking, galvanic stimulation results in deviations of the heading direction towards the side of the anode [27]. Similarly, unilateral damage to the vestibular apparatus results in heading deviations towards the side of the lesion, particularly during slow walking [88,112]. Galvanic vestibular stimulation also provides the opportunity to alter vestibular afferent firing during particular phases of the step cycle. The effects on the gait are largest when stimulation is initiated at heel contact and minimized during the swing phase [113], and could contribute to a role of the vestibular system in the planning of future foot placement for forward progression [113,114], with the current swing phase being coordinated by local spinal circuits. Interestingly, this phase-dependent role of the vestibular system was only present in the limbs, whereas control of the upper body was independent of the step cycle, perhaps indicating separate control systems for the maintenance of posture and locomotion. Interestingly, vestibular stimulation appears to have less effect on gait direction and variability when running compared to walking [115].

Vestibular damage also results in gait variability [89], a potential phenotype that may not be directly related to an inability to balance or poor postural control. This could indicate that vestibular feedback can have a role in foot placement, perhaps due to the requirement of the vestibular system for understanding the position of the body in space [116]. Temporal gait variability is associated with damage to both the vestibular system and the cerebellum. Interestingly, variability associated with cerebellar damage manifests at both slow and fast walking speeds, whereas the variability found in vestibular patients is only observed at slow speeds, with a normal variance found at higher gait speeds [89]. This indicates that the role of the vestibular system in locomotion may be speed-dependent, which is further discussed below.

### 3.5. Locomotor Speed and Vestibular Influence

In general, vestibular damage results in a slower gait speed [117], at least partly due to patients taking longer, slower steps when walking [118]. As discussed above, this slow gait is highly variable both in the temporal and spatial domains [119]. Intuitively, there could be two potential reasons for this. First, the vestibular system is believed to be involved in setting the desired pace of locomotion, and vestibular stimulation can result in an increase in gait speed [120]. Therefore, vestibular damage may result in a distorted perception of locomotor speed. Second, a general feeling of disequilibrium or instability could simply result in the nervous system, reducing locomotor speed to protect the body from falls. The reduced locomotor speeds would increase the duty cycle; therefore, there is a higher number of legs providing support. This, in turn, could mean a higher rate of somatosensory feedback due to longer ground contact (cutaneous feedback) or load signals (group Ib feedback from the Golgi tendon organs).

However, several studies have noted that gait variability resulting from vestibular damage is reduced when the locomotor speed is increased. As mentioned, gait variability is reduced in patients with bilateral vestibular failure during fast walking but not in patients suffering from cerebellar ataxia [89]. Animals with vestibular damage that are unable to walk in straight lines when walking are capable of maintaining a constant heading direction when running [88]. Similarly, humans with vestibular neuritis are able to maintain a constant heading when running slowly but not when walking [88]. This same study suggested that these differences could be explained in the differences between spinal vs. the descending control of locomotion [88], with the largely spinal high-speed locomotion prompting an inhibition of the descending pathways carrying vestibular information [115].

The potential speed dependence of vestibular signaling provides some important clues as to the relative roles of vestibular and proprioceptive feedback during locomotion. We discuss these below.

## 4. Perspective

Locomotor behavior is controlled by interactions between the CPG, sensory feedback from the segmental somatosensory system, as well as from supraspinal sensory input, which includes the vestibular system. The control of normal locomotion requires the interactions of multiple sensory systems. Indeed, both vestibular and somatosensory signals can be found in similar brain regions, particularly cortical regions [121], and vestibular processing can be influenced by somatosensory signals [122]. However, the role and way that segmental somatosensory feedback and the vestibular system affect the function of the CPG is very likely to be distinct in three ways. First, while somatosensory feedback has a patterning effect for each locomotor cycle, vestibular feedback seems to have a more subtle, indirect influence on locomotion, unless the locomotion is perturbed. Second, the availability of vestibular influence on locomotion appears to be state-dependent, whereas somatosensory feedback is available throughout the locomotion. Though it is worth noting that some specific reflex pathways are reduced starting just before the initiation of movement or even reversed in sign during locomotion compared to the rest [123,124]. Third, the influence of segmental somatosensory feedback and vestibular feedback on locomotor behavior seems to be speed-dependent, such that segmental feedback is necessary at higher speeds, whereas vestibular feedback is required for slower speeds [11,88]. In the following, these three aspects will be discussed separately.

### 4.1. Effect of Segmental Somatosensory and Vestibular Feedback on the Generation of Locomotion

It has been established that the locomotor pattern driving well-coordinated locomotor behavior is generated by the interactive function of the CPG and sensory feedback [2]. We discussed above that segmental somatosensory feedback does influence very specific aspects of locomotor movements transiently on a cycle-to-cycle basis, likely through the direct and specific influence of the CPG network. This influence seems to be important during unperturbed locomotion [2,8,74,125], as well as to compensate for mechanical perturbations [8,55,126]. In contrast, vestibular pathways seem to play several accessory roles in locomotion; most of these can be explained by the need to maintain an upright posture when walking. Potentially, the vestibular system does not directly influence the CPG but, rather, has a more general influence on the overall function of the CPG when the animal is performing a smooth, undisturbed locomotion. However, when locomotion is perturbed, such as a sudden lateral movement of the terrain, supraspinal pathways influenced by the vestibular system can step in to provide the necessary motor program that enables the animal to perform corrective movements [127]. From this, it seems that both segmental somatosensory feedback, as well as vestibular feedback, are important for the generation of a functional locomotor pattern, but the way these feedbacks are utilized is different.

### 4.2. State-Dependent Modulation of Segmental Somatosensory and Vestibular Feedback

Segmental somatosensory feedback is modulated in state and phase-dependent manners [128], but the feedback is always available to the nervous system so that ongoing locomotor behavior can be modified in different terrains. This is different from vestibular feedback. Vestibular feedback is the key to maintaining posture and avoiding moving/swaying during standing. It is counterintuitive at first glance for the vestibular system, functioning as a stabilizer to keep us in place, to have a function during locomotion, which is defined as moving from one place to another and is inherently unstable. However, it was shown that the vestibular feedback is downregulated at the transition to locomotion and has different effects during different parts of the step cycle [107,113], at least in humans. This suggests that vestibular feedback is available during locomotion and can be transitorily called upon at different points of the step cycle to ensure that locomotion does not disrupt the upright posture. This ability likely requires the nervous system to integrate both current vestibular sensory feedback, as well make feedforward predictions of how locomotor actions will impact the postural stability. This integration of sensory feedback and feedforward predictions likely underlies natural locomotion and could involve other supraspinal structures, such as the cerebellum [129].

### 4.3. Influence of Segmental Somatosensory and Vestibular Feedback at Different Locomotor Speeds

Even though segmental sensory feedback is important for the generation of a normal locomotor behavior, its relative necessity seems to be speed-dependent. It has been shown that animals, including humans, can perform locomotion if segmental feedback is partially removed, even though specific changes occur in the timing and amplitude of the motor activity [74,125]. However, if the segmental proprioceptive feedback is completely removed, the movements become maladaptive [74]. Moreover, in animal models with proprioceptive sensory feedback removed from the muscle spindles, locomotion remains at slower speeds, with higher locomotor speeds avoided [11]. Interestingly, there seems to be a reverse relationship with the vestibular feedback and locomotor speeds. That is, humans and animals with vestibular damage prefer a higher locomotor speed [88]. These observations indicate that, whereas segmental somatosensory feedback, especially proprioceptive feedback, is required for higher speeds, vestibular feedback is necessary for lower speeds. However, these observations do not address the question why this would be the case, nor what the underlying circuit mechanism are. It is conceivable that higher speeds are prevented due to issues related to biomechanics or spinal circuit functions. Addressing this will require further investigation.

### 4.4. Current Working Hypothesis

Based on the above, we hypothesize: “the role of segmental and vestibular feedback during locomotion depends on speed, vestibular feedback is required at lower speeds while the somatosensory feedback is necessary at higher locomotor speeds.” The rational for this hypothesis is the following:

(i). Slower locomotor speeds are more variable and unstable than higher speeds. As slower walking speeds are more likely to be associated with exploratory activities, they require frequent changes in the heading direction and body position. Here, the nervous system must consider how each movement or variation in foot placement could affect the equilibrium. Therefore, a direct link to the head movement, and overall position of the body, is imperative.
(ii). Due to the slower speed of movement, there is ample time for the brain to influence the spinal networks to control foot placement and posture. Multiple descending pathways influence locomotion, with the vestibular system “in charge” of making sure that these movements do not cause the animal to lose balance.
(iii). During walking, the features of the step cycle, such as overall cycle duration or length of the support phase, are variable at different walking speeds, whereas the timing of these phases during running is consistent across speeds [130]. High-speed locomotion could therefore be more stereotyped and perhaps dominated by local spinal networks where segmental somatosensory feedback is the main source of sensory feedback, allowing minor adaptations to the musculature as locomotion continues.
(iv). The segmental proprioceptive feedback seems to be important for all speeds, it is necessary for higher speeds, as, without them, animals do not locomote at higher speeds.

## 5. Future Perspective in the Age of Molecular Sciences

Clearly, locomotion is a complex behavior requiring multiple modalities of sensory feedback, as well as feedforward predictions from the brain. How are we to untangle this complexity of different neural circuits? Traditionally, the identification of the neural circuitry that facilitates locomotion has relied on the electrophysiological mapping of neurons in the spinal cord, sensory pathways and brain [131,132]. Furthermore, many of the studies discussed above have been based during observations of human patients, precluding a detailed analysis of the underlying circuitry. As we mentioned in the introduction, the mouse presents an important opportunity to dissect the neural circuitry underlying locomotion. Mouse genetics can be used in two broad ways to target neural circuitry. First, knockout strategies can be used to remove individual genes in neurons. The resultant phenotype can be observed, and some conclusions can be drawn around the role of the underlying circuit changes. Genetic knockouts can be either global, i.e., the simple removal of a gene from the entire organism, or conditional, where the gene is removed only from select tissues or cell types. Further information on the use of gene knockouts in neuroscience studies can be found elsewhere [133]. As well as traditional gene knockouts, in the future, more contemporary technologies that use gene editing, such as CRISPR–Cas systems, will also be highly important for understanding the roles of particular circuits in motor behaviors [134].

**Figure 2:**
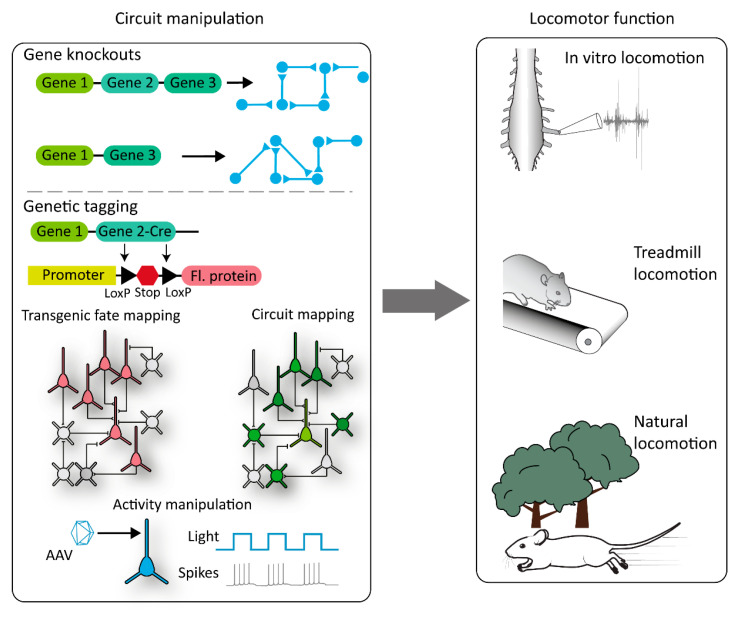
Summary of the molecular genetic strategies that can be used to dissect the locomotor circuitry. Gene knockouts (top line) can lead to circuit rearrangements that can be combined with several techniques to analyze the function of that circuit (right). Similarly, gene expression can be used to tag populations of neurons and probe their functions via manipulations of their activity or by tracing their synaptic inputs. Fl. protein = fluorescent protein.

An important example using traditional genetic knockouts can be found in the manipulation of axon guidance molecules that guide axons to their targets during development, such as the genetic deletion of the EPhA4 receptor and resultant phenotype of the synchronous left–right “hopping” gait [135,136]. This genetic strategy has led to an increased understanding of the role of commissural interneurons in the coordination of left–right alternations [137] (Figure 2).

Complimentary strategies utilize gene expression patterns in specific subsets of neurons, but rather than probe the function of that gene directly, they use the expression as a “marker” for that subtype and target genetic or viral tools to probe the circuit function. In the spinal CPG, the most common strategy has been to utilize the wealth of knowledge we have regarding transcription factor expression during development and the consequent sorting of spinal interneurons into four cardinal domains (V0–V4) [5,138]. These classes can be further subdivided into more genetically and anatomically neuronal classes [139,140]. A common strategy is to “fate map” developing spinal interneurons and express proteins in adult animals that can either alter their function or remove them from the circuit completely (Figure 2). Fate mapping generally involves the use of two separate mouse lines bred together to produce progeny where select tissues or cell types permanently express a transgene (for example, a fluorescent protein). One mouse line contains a site-specific recombinase (such as cre) inserted downstream of the gene of interest, and the expression of the recombinase will therefore be tied to that gene’s appearance. The second mouse line contains an exogenous gene, such as a fluorescent protein, inserted into the genome under the control of a ubiquitous promoter. Importantly, this gene will be downstream from a transcriptional stop flanked by LoxP sites (in the case of cre recombinase). Under wildtype conditions, the fluorescent protein is not expressed. When both cre and this reporter construct are in the same cell, the transcriptional stop is permanently removed. As this removal is not reversible, the fluorescent protein will continue to be expressed even after the gene of interest has been downregulated. This makes fate mapping an important strategy to study developmentally regulated genes, where exogenous genes can be expressed in the adult dependent on their developmental gene expression profile. Further information on the use of fate mapping can be found in a different review [141].

These strategies have yielded important information regarding the function of these broad classes of neurons, such as V1 interneuron involvement in locomotor speed [142]. Finally, through the introduction of genetic recombinases, gene expressions in adult spinal neurons can be exploited to target genetic actuators of distinct cells types. These strategies can be used to manipulate the function of spinal neurons—for example, with chemo- or optogenetics—or reveal neuronal inputs with transsynaptic tracers, such as the rabies virus (Figure 2). An important example of this was the use of Chx10 cre lines to probe the function of V3-derived neurons in both the brain and spinal cord [143,144,145].

Despite the common use of genetic strategies to dissect the CPG circuitry, the use of the same tools to dissect sensory contributions to locomotion such as the vestibular and proprioceptive systems has been relatively lacking. These pathways, though, are amenable to the same types of mapping and manipulation strategies. For example, rabies virus tracing can be utilized to dissect the neuronal outputs of vestibular and proprioceptive sensory neurons [146]. Genetic knockout strategies have also been used to pinpoint the role of proprioceptive neurons in locomotion, such as the use of Egr3-mutant mice to investigate the role of proprioceptive sensory feedback in locomotion [74], while a combination of genetic markers and viral manipulations have been used to remove proprioceptive feedback from select muscle groups [11].

Compared to other sensory pathways, such as the sensory neurons involved pain or touch [35,36] or the auditory system [147], our understanding of the genetic subclasses of the proprioceptive and vestibular pathways is relatively limited. However, important progress is being made in these pathways. The subtypes of proprioceptive neurons can be subdivided based on their expression of the ETS transcription factor [148], and the combinatorial expression pattern of various markers can be used to target phenotypically distinct proprioceptive neurons [149]. Similarly, in the vestibular system, the developmental pathways that delineate both vestibular sensory neurons and vestibulospinal neurons are known [150,151], although there remains much work to do. A continuing push towards a genetic understanding of the neuronal subtypes in the proprioceptive and vestibular circuitry, combined with modern molecular genetic strategies, will result in a more complete understanding of these sensory pathways roles in movement.
